# Supplementary material for: CX3CL1 homo-oligomerization drives cell-to-cell adherence
Source: Sci Rep. 2020 Jun 3;10:9069. doi: 10.1038/s41598-020-65988-w (PMC7271195; doi:10.1038/s41598-020-65988-w)
Supplement: Supplementary file 1 — Supplementary information. [file 41598_2020_65988_MOESM1_ESM.pdf]

Supplementary Information

CX3CL1 homo-oligomerization drives cell-to-cell adherence

Mariano A. OSTUNI[1*], Patricia HERMAND[1*], Emeline SAINDOY[1§], Noëlline GUILLOU[1], Julie GUELLEC[1†], Audrey COENS[1‡], Claude HATTAB[2], Elodie DESUZINGES-MANDON[3], Anass JAWHARI[3], Soria IATMANEN-HARBI[4], Olivier LEQUIN[4], Patrick FUCHS[4,5], Jean-Jacques LACAPERE[4], Christophe COMBADIÈRE[1], Frédéric PINCET[6] and Philippe DETERRE[1]

[1]Sorbonne Université, Inserm, CNRS, Centre d'Immunologie et des Maladies Infectieuses (CIMI-Paris), U1135, F-75013, Paris, France

[2]Université de Paris, Institut National de la Transfusion Sanguine, INSERM, UMRS 1134, F-75015 Paris, France

[3]CALIXAR, Bâtiment Laennec, 60 avenue Rockefeller, F-69008 Lyon, France

[4]Sorbonne Université, Ecole Normale Supérieure, PSL Research University, CNRS, Laboratoire des Biomolécules (LBM), F-75005 Paris, France

[5]Université de Paris, UFR Sciences du Vivant, F-75013 Paris, France

[6]Sorbonne Université, Ecole Normale Supérieure, PSL Research University, CNRS, Université Sorbonne Paris Cité, Laboratoire de Physique, F-75005 Paris, France

[*]Present address: Biologie Intégrée du Globule Rouge, UMR_S1134 ; Institut National de la Transfusion Sanguine ; 6, rue Alexandre Cabanel ; 75015 Paris, France

[†]Present address: Univ Brest, Inserm, EFS, UMR 1078, GGB, F-29200 Brest, France

[‡]Present address: I2BC, CEA Saclay, Bât 144, F-91191 Gif-sur-Yvette, France

[§]Present address: Centre de Recherche Saint Antoine, 27 Rue de Chaligny F-75012 Paris, France

Corresponding author:
Philippe Deterre, CIMI-Paris, 91, boulevard de l'Hôpital, F-75013, Paris, France, philippe.deterre@upmc.fr, ORCID iD: 0000-0001-9303-0791

Table S1
^{1}H chemical shift assignments of TM24 in DPC (500 MHz, 40°C)

Residue	HN	Hα	Hβ	Other side chain H
Lys1	-	4.04	1.91	
Lys2	8.92	4.33	1.83	Hγ 1.47, 1.40 Hδ 1.68
Val3	8.11	3.83	2.09	Hγ 0.95, 0.89
Gly4	8.44	3.88, 3.83		
Leu5	8.07	4.10	1.70	Hδ 0.96, 0.89
Leu6	8.20	3.97	1.85, 1.49	Hγ 1.79 Hδ 0.89, 0.82
Ala7	8.26	3.94	1.41	
Phe8	7.94	4.14	3.21, 3.14	Hδ 7.04
Leu9	8.33	3.80	1.87, 1.44	Hδ 0.80, 0.59
Gly10	8.56	3.63, 3.50		
Leu11	8.22	3.94	1.77, 1.65	
Leu12	8.04	3.75	1.60, 1.35	Hγ 1.41 Hδ 0.80, 0.59
Phe13	8.45	4.03	3.22, 3.06	Hδ 7.03 Hϵ 6.96 Hζ 6.89
Cys14	8.16	3.80	3.15, 2.69	Hγ 2.50
Leu15	8.39	3.89	1.75	Hδ 0.74
Gly16	8.58	3.61, 3.48		
Val17	8.36	3.48	1.98	Hγ 0.80, 0.60
Ala18	8.40	3.96	1.47	
Met19	8.50	4.08	2.15, 1.97	Hγ 2.52, 2.41
Phe20	8.27	4.18	3.15, 3.04	Hδ 7.20
Thr21	8.02	4.08	3.90	Hγ2 1.03
Tyr22	7.96	4.33	3.05, 2.91	Hδ 7.05 Hϵ 6.70
Lys23	7.70	4.12	1.81, 1.72	Hγ 1.35 Hδ 1.59
Lys24	7.74	4.03	1.70, 1.62	Hγ 1.29 Hδ 1.56 Hϵ 2.88 Hζ 7.61

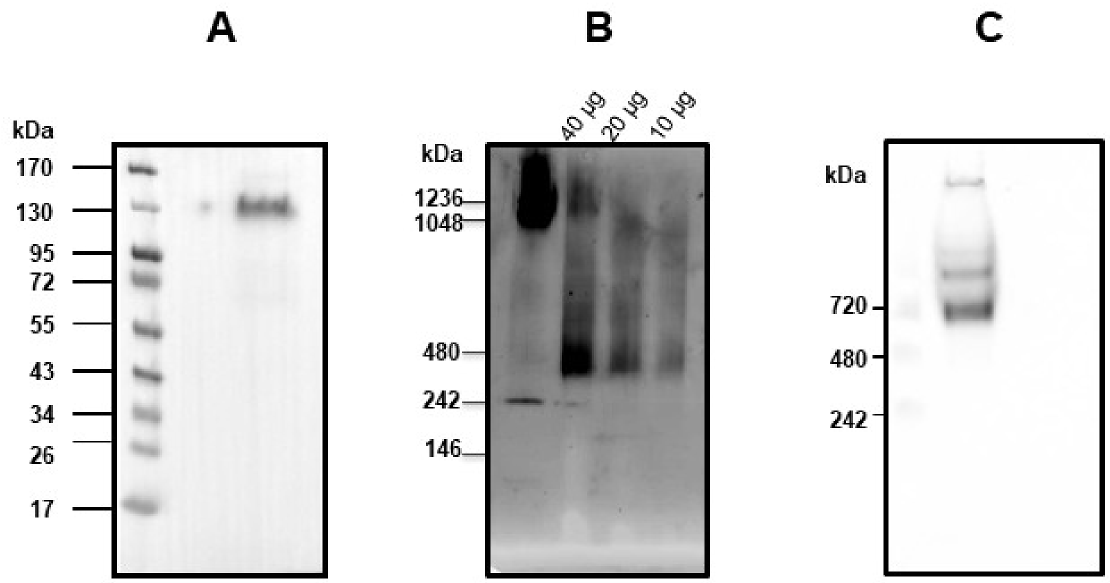

Fig S1

Electrophoresis of the CX3CL1-YFP fusion protein

A. Cell lysate of L_{CX3CL1} cells stably transfected with CX3CL1-EYFP was analyzed by SDS-PAGE and Western blot (full length blot corresponding to Fig 1A)
B. Cell lysates (containing 40, 20 and 10µg of total proteins) of L_{CX3CL1} was analyzed by native electrophoresis using Nu-Page gel in the presence of 1% DDM (dodecylmaltoside) and Western blot (full length blot corresponding to Fig 1B)
C. The affinity-purified CX3CL1 from L_{CX3CL1} membranes was analyzed by native PAGE. CXCL3 was solubilized using CALX173ACE and affinity purified (CXCL1 antibody). Mini-proteon TGX gel electrophoresis (Biorad) were used for Native PAGE(full length blot corresponding to Fig 1C)

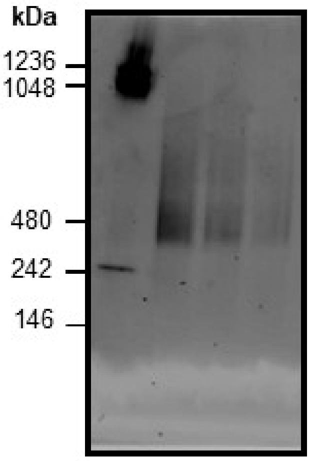

Fig S2

Native gel electrophoresis of the CX3CL1-YFP fusion protein using digitonin

Cell lysates (containing 40, 20 and 10µg of total proteins) of L_{CX3CL1} were analyzed by native electrophoresis using Nu-Page gel in the presence of 1% digitonin and Western blot.

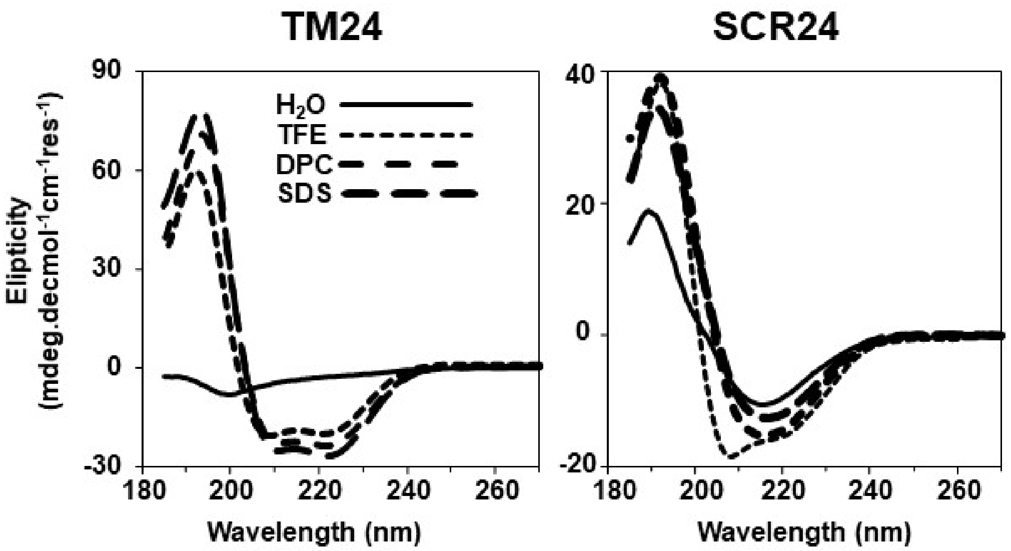

	Hel	Bet	Turn	Des
H_2O	7	31	24	38
TFE	85	2	3	10
DPC	94	1	0	5
SDS	96	0	0	5

Hel	Bet	Turn	Des
7	29	25	38
75	2	7	17
66	8	8	19
55	12	12	22

Fig S3
Circular dichroism of FKN24 and SCR

CD spectra recorded in H_2O (continuous line) show that both peptides are poorly ordered whereas they exhibit high helical content in TFE (dashed line), a well-known solvent that favors helices formation; SDS and DPC, detergents that form micelles, also favors helical structure. The presence of L close to both ends of the SCR24 peptide might contribute to the lower helical content compare with TM24.

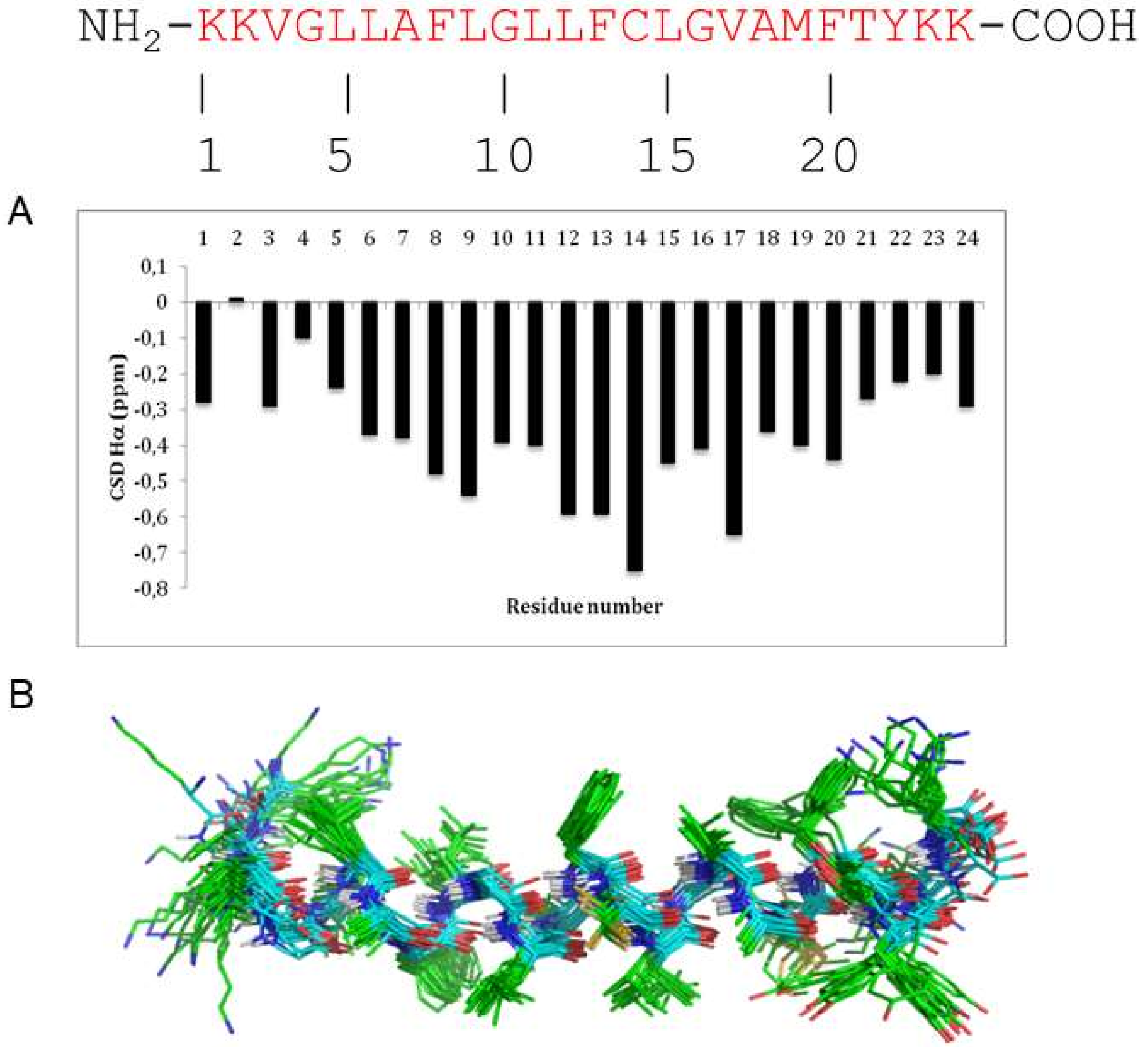

Fig S4

NMR conformational study of TM24 in DPC micelles.

A. Chemical shifts deviations (CSDs) of Hα protons with respect to random coil values. The large negative chemical shift deviations (CSDs < –0.1 ppm) indicate mainly helical conformations for segment Val3-Lys24.

B. Family of the best 20 NMR structures. Structures were superimposed for best fit of backbone N, Cα and C' atoms. The structure family was drawn with Pymol program (DeLano, W. L. The PyMOL Molecular Graphics System; DeLano Scientific: San Carlos, CA (2002))

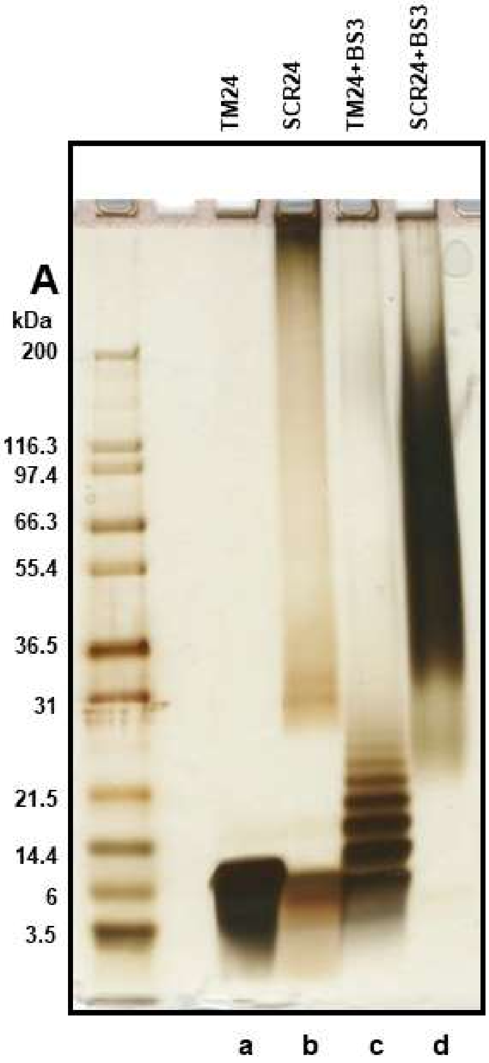

Fig S5

TM24 peptide polymerization analyzed by cross-linking and SDS-PAGE

SDS-PAGE of the TM24 and SCR24 peptides dissolved in DPC with a ratio [DPC]/[peptide] = 10 and cross-linked or not with 1.9 mM SB3 crosslinker. The gel is then silver stained. The left lane contains markers of various molecular weights (full length gel corresponding to Fig.4A)

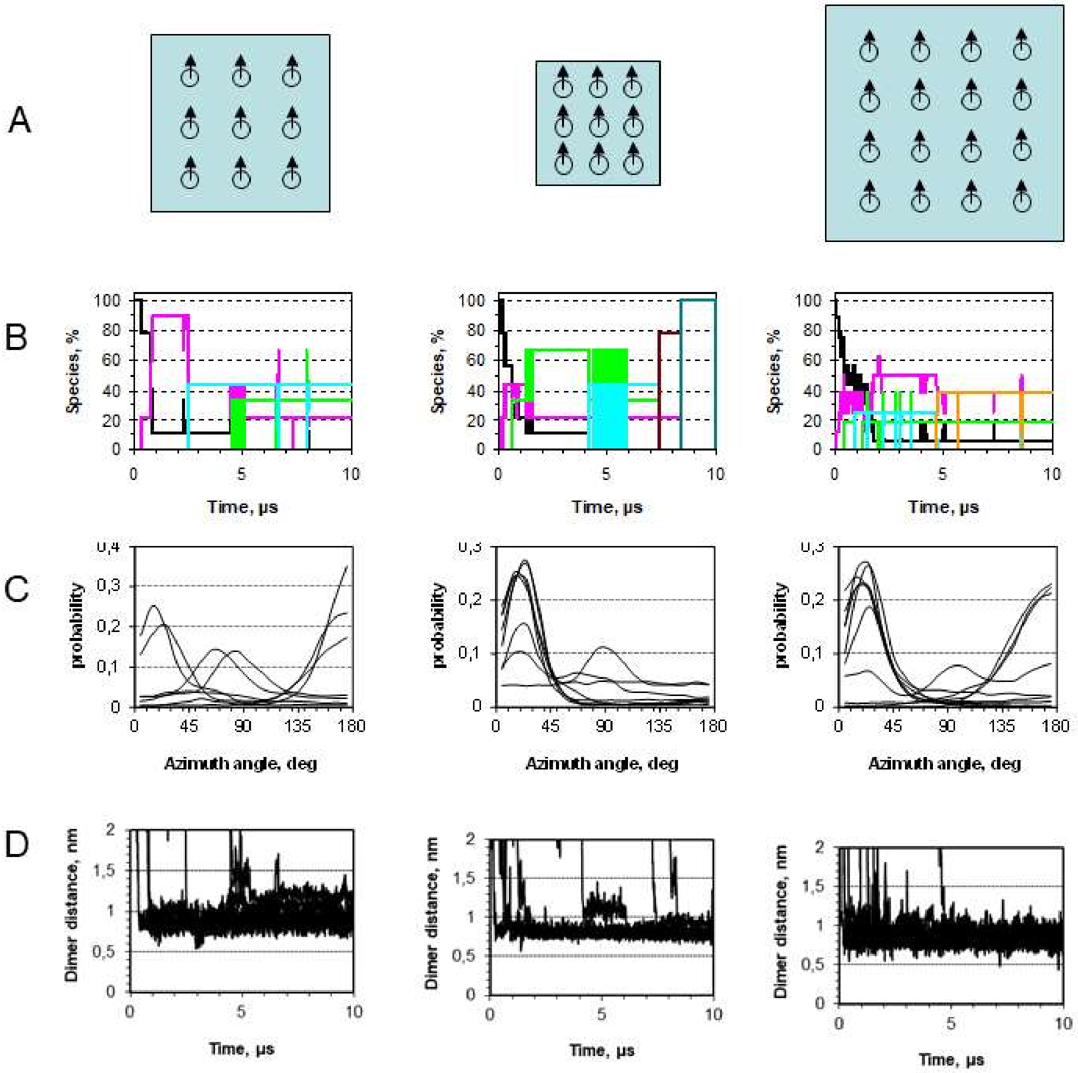

Fig S6

Coarse-grained molecular dynamics simulations of TM24 in DOPC lipids.

A. Scheme of the three TM24/DOPC boxes built at molar ratios of 9/900, 9/450 and 16/1600 from left to right, respectively.

B. Oligomer formation as a function of time for the various boxes showing the population of the different species. Black trace corresponds to monomer, purple to dimer, cyan to trimer, green to tetramer, orange to pentamer, brown to heptamer and dark green to nonamer.

C. Probability distribution of the azimuthal angle between the two helix pair in each dimer. This latter is defined as the angle between two vectors (one for each helix) going from the backbone bead of Leu11 to that of Phe13 (those two residues are roughly opposite on the helical wheel of TM24) projected on the bilayer plane. Three populations can be observed, the main one corresponding to a nearly parallel arrangement with an angle of 20-30 degrees.

D. Center of mass-center of mass distance between each pair of interacting helices as a function of simulation time. Most of the arrangements exhibit an average distance of 0.8+/-0.1nm. Observed spikes correspond to transient and reversible separations during which each helix can freely rotate about its axis.

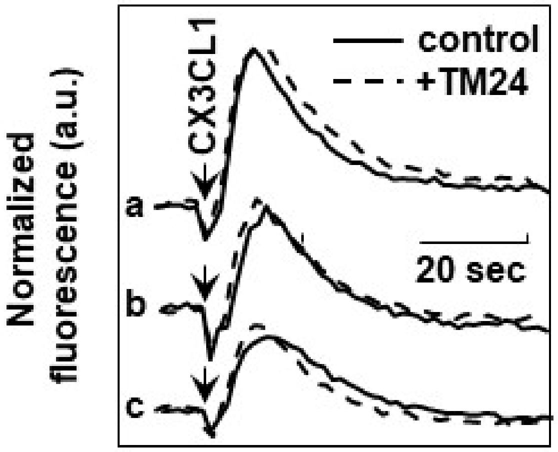

Fig S7

Calcium response of CHOCX3CR1 to soluble CX3CL1 in the presence TM24

The calcium response of CHO_{CX3CR1} cells to 200 nM (a), 100 nM (b) and 50 nM (c) soluble CX3CL1 (Chemokine Domain) were performed using CalbryteTM520 fluorescent marker in the presence (dashed traces) or absence (solid traces) of 5µM of the TM 24 peptide. The data were the mean of triplicates.

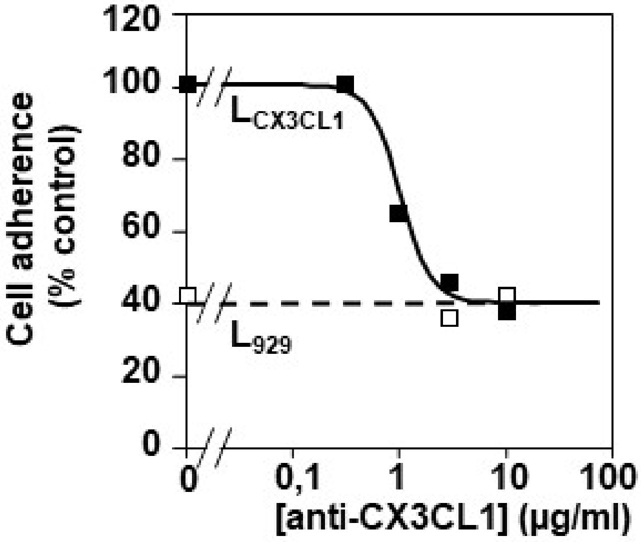

Fig S8: Inhibition of the CHO_{CX3CR1} adherence to L_{CX3CL1} by AF365 clone of anti-CX3CL1 antibodies

Adherence of CHO_{CX3CR1} cells to L_{929} (empty squares, dashed trace) or L_{CX3CL1} cells (filled squares, solid trace) as determined by the LigandTracer^{TM} technique 60 minutes after addition of CHO_{CX3CR1} cells, in the presence of various concentrations of the anti-CX3CL1 antibody. The data were normalized using the control trace with L_{CX3CL1} cells in the absence of antibody. Each point was the mean of duplicates.
